# Enhancing Cross-Cultural Competence of Medical and Healthcare Students with the Use of Simulated Patients—A Systematic Review

**DOI:** 10.3390/ijerph20032505

**Published:** 2023-01-31

**Authors:** Aleksandra Walkowska, Piotr Przymuszała, Patrycja Marciniak-Stępak, Maria Nowosadko, Ewa Baum

**Affiliations:** 1 Centre for Foreign Language Tuition, Poznan University of Medical Sciences, 60-801 Poznan, Poland; 2 Department of Medical Education, Poznan University of Medical Sciences, 60-806 Poznan, Poland; 3 Department of Medical Simulation, Poznan University of Medical Sciences, 60-806 Poznan, Poland; 4 Department of Social Sciences and the Humanities, Poznan University of Medical Sciences, 60-806 Poznan, Poland

**Keywords:** cultural competence, healthcare students, simulations, simulated patients, diverse patients, cross-cultural education

## Abstract

Increasing cultural and linguistic diversities of populations have created a challenge for medical educators to provide authentic learning experiences fostering cross-cultural understanding and interprofessional attitudes of students. Simulations with actors portraying patients (commonly referred to as simulated patients) are effective learning modalities to teach students to provide culturally competent care and influence the quality of patient-centered care. The aim of this systematic review was to identify and synthesize available evidence on the use of simulations with simulated patients as a learning intervention to teach cultural competence to the students of healthcare professions. The PubMed, Medline Complete, and CINAHL databases were searched for articles, which resulted in 27 papers being included in the review. Results revealed that engaging students in cross-cultural interactions with patients increases their level of cultural competence, confidence, and learning satisfaction, and therefore, simulations with simulated patients can serve as a powerful reinforcement of cross-cultural education.

## 1. Introduction

The term ‘culture’ denotes beliefs, values, norms, and lifeways that can be shared, learned, and passed on among individuals or specific groups of people [1]. Culture exerts influence on the way people think, judge, or make everyday decisions. Cultural competence involves a gradual process of development of healthcare professionals’ capacity “to provide safe and quality healthcare to clients of different cultural backgrounds” [1]. Cultural competence is considered an integral part of contemporary medical education, with the main objective of ensuring that people from culturally and linguistically diverse environments receive the best quality care. Doctors, nurses, and other healthcare providers are expected to take into consideration their patients’ values, beliefs, behavioral patterns, and linguistic needs to deliver culturally competent care [2]. It seems necessary to educate future healthcare professionals about factors affecting the health of under-represented patient populations, including their understanding of treatment strategies, ability to recognize symptoms, healthcare needs, social isolation, accessibility of health services, economic stability and employment chances, immigration and insurance status, education, various customs and cultural patterns, religious influences, family support, decision-making patterns, fear of discrimination, communication styles, language barriers and non-verbal attributes of communication to ensure the safety of treatment by enhancing access to the services and treatment compliance [3,4,5,6,7,8,9,10,11,12,13]. The necessity to prepare doctors, nurses, and other healthcare workers to provide safe and culturally competent care was noticed by policy-makers around the world. For instance, the US Department of Health and Human Services advocates medical educators to include cultural and linguistic competence into teaching curricula to enhance awareness of the importance of culture and language in the process of healthcare delivery [14]. The International Council of Nurses, a federation of more than 130 national nurses associations representing millions of nurses worldwide, states that nurses ought to be culturally and linguistically competent to provide appropriate and effective care to patients regardless of their cultural or linguistic backgrounds [15]. Additionally, in Poland, the Ordinance of the Ministry of Science and Higher Education on the standards of education for the faculties of medicine, dentistry, pharmacy, nursing, midwifery, medical analytics, physiotherapy, and medical rescue, which regulates learning outcomes intended to be achieved by the students, stresses the importance of teaching about acceptance, understanding, and respect towards a culturally diverse patient and underlines the need of building awareness of cultural and ethnic differences [16]. The role of language skills, which are an indispensable element of cultural competence as culture influences language and communication [17], is also appreciated by including obligatory second language learning into the curricula and advocating the acquisition of minimum B2 level (CEFR) of its competence by the graduates [16]. This seems especially relevant in view of the fact that language may act as a barrier to effective communication and an obstacle to accurate patient–doctor interaction, undermining the patient-centered approach to culturally competent care [18].

A wide variety of training methods is used to teach cultural competence, including lectures, tutorials, class debates, discussions, cultural immersions with local and international communities, watching videos, storytelling, student group presentations, case scenarios, question-and-answer sessions, reflective feedback, role-plays, or simulation methods [19,20]. The last one may take various forms and involve the use of life-size mannequins or actors trained to perform the role of the patient, often referred to as simulated or standardized patients. The use of simulation methods has numerous advantages as they give a learner a chance to engage in clinical encounters and practice their skills without putting real patients’ safety at risk [21]. They also lower students’ anxiety by giving them an opportunity to gain experience in a non-threatening environment, increase confidence, help in understanding the role of culture in healthcare due to the introduction of diverse cultural attributes, and allow them to apply theoretical knowledge into practice [21]. Moreover, debriefing after each scenario helps the trainees to understand the learning goals through reflection, feedback, and discussion [22,23]. Finally, the preferences of trainees for active case-based behavioral simulations rather than passive teaching strategies have been emphasized [19].

The use of ‘actors trained to play patients, which enables training and assessment of history taking, physical examination, communication skills, and professionalism’ [23] has proved a valuable learning modality, well documented in the literature and first reported by H. Barrows, a neurologist, who introduced them in the 1960s [24]. They are usually efficiently trained to be able to simulate various medical conditions in a realistic and authentic way [25]. However, it should be noticed that despite the popularity of the method, the literature on their nomenclature remains inconsistent. While some authors do not see differences between standardized and simulated patients, other researchers notice some distinctions between them [26,27,28]. For some researchers, standardized patients denote individuals presenting their own symptoms, history, or character consistently and in the same way [26], while others consider standardized patients to constitute a broader term, including simulated and adequately trained real patients [29,30]. Given the ambiguity described above and the postulated need to unify the nomenclature, throughout this paper, we use the term ‘simulated patient’ (SP) in the meaning defined by H. Barrows, who sees them as individuals who were rigorously trained to simulate a real patient, including symptoms [29].

All in all, simulated and standardized patients are believed to bring realism to a simulation experience and, among others, increase students’ attitudes and self-efficacy in dealing with problems that may arise in a real-life clinical situation [31]. They allow for building on cultural content during a simulated clinical event by incorporating into the scenario clothes, wigs, ethnic-inspired jewelry, religious artifacts, culturally specific names, language, eye contact, mannerism, and personal space that are consistent with the culture illustrated in the simulated situation [32,33]. Most of all, simulations with human actors create a psychological challenge, give a possibility to experience emotions, and prepare students in safe conditions to manage stress in future real-life clinical events [34]. Finally, the educational aspect of simulations with SP is emphasized by the feedback from the patient, who has been professionally trained on how to evaluate students’ performance in a supportive and encouraging manner [35]. However, there are also some barriers to the standardized simulation program. Rutledge et al. named the costs, which involve the actors’ fees and training, access to actors, which can be especially difficult when patients from various cultural and ethnic groups need to be recruited to ensure realistic cultural encounters and anxiety and intimidation of the students facing an encounter with an SP, particularly when they are not familiar with the teaching concept [36]. 

Considering all of the above, the aim of this review was to identify and analyze the literature evidence on the role of the SP simulation method in the process of cross-cultural competence training of healthcare students.

## 2. Materials and Methods

### 2.1. Study Selection and Inclusion Process

**Figure 1:**
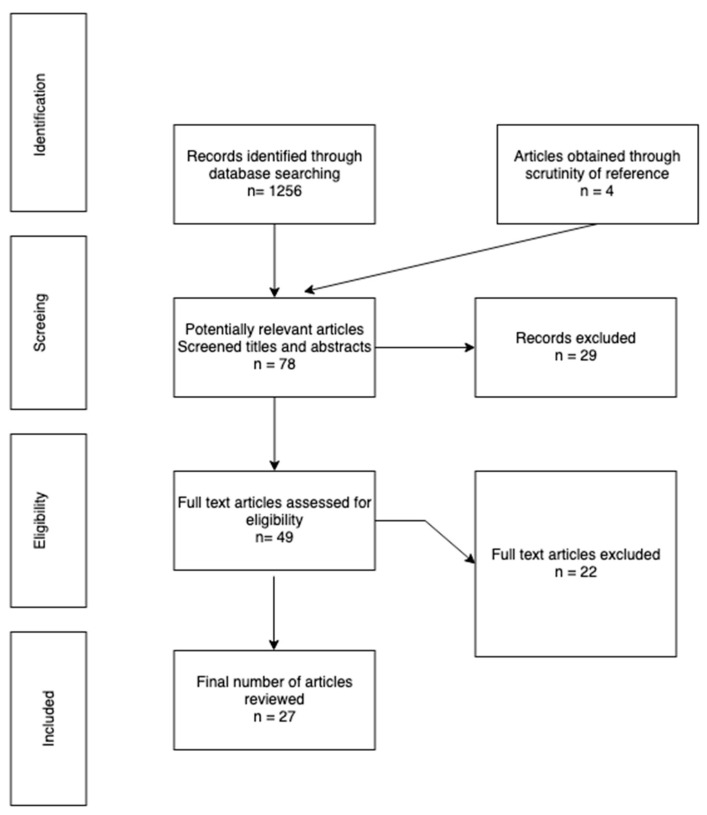
Study selection and exclusion process.

A literature review was carried out to analyze studies on the implementation of simulated patients to provide cultural competence education to healthcare students. The articles were acquired from PubMed, Medline Complete, and CINAHL databases, using keywords of ‘simulation and cultural competence’, ‘standardized patient and transcultural education’, ‘cross-cultural training and simulation’, as well as the following Boolean combination: ((simulated patient*) OR (standardized patient*) OR (simulation*)) AND (student*) AND ((multicultural*) OR (cross-cultural*) OR (transcultural*) OR (cultural*)) (Figure 1). The databases were searched between February and July 2022. No limitations were introduced in terms of the publication year. 

### 2.2. Inclusion/Exclusion Criteria

The search was limited to articles written in English, which discussed cultural competence education conducted with simulated patients among healthcare professionals during undergraduate programs. The authors disqualified publications that did not cover the subject of the simulated patient method as the tool for training in cross-cultural competency. Mannequins with human voices, students engaging in role-plays, virtual reality, simulation games, or pre-recorded videos were not included in the research. The detailed systemic review questions, along with applied inclusion and exclusion criteria, are provided in Table 1.

The primary search of databases resulted in 1256 identified article records, and 4 further papers were identified through manual search from reference lists. The initial screening of the titles, followed by an abstract screening in terms of inclusion and exclusion criteria of 78 potentially relevant papers, yielded 49 results for full-text analysis. These papers were analyzed and assessed for eligibility, which led to the inclusion of 27 articles and the exclusion of 22 articles, which, upon in-depth reading, did not comply with the inclusion criteria (Table 1). The analysis and selection were performed by two independent researchers, who systematically reflected on the process in discussions and consultations. 

## 3. Results

### 3.1. Participants of Studies

In total, 27 studies that matched the inclusion criteria were identified. The studies were conducted in five countries: twenty-two in the United States [4,5,6,7,11,12,33,35,37,38,39,40,41,42,43,44,45,46,47,48,49,50], two in Australia [9,51], two in Turkey [52,53], one in Canada [54] and Norway [33]. Among them, one study was a bi-national intervention carried out at both American and Norwegian universities [33]. Six studies were conducted on medical students [5,37,38,39,40,54]. Twelve articles focused on nursing students [6,7,11,12,33,41,42,43,49,50,52,53]. Three papers presented data on dentistry students [4,35,46], one on pharmacy students [47], and one on physiotherapy students [48]. Four of the studies used mixed-group participants from different healthcare faculties [9,44,45,51]. 

### 3.2. Studies on Medical Students

As disclosed above, six identified studies were conducted on medical students, involving two articles presenting results from the same intervention [5,39]. Their sample sizes ranged from 22 to 197 students. Most of the papers presented the results of post-intervention measures [5,37,39,40,54], with one using the pre–post study design [38]. Two of the studies presented the intervention during a cultural content OSCE station [5,39] and one carried out tOSCE [38], which is a formative assessment method, as opposed to summative OSCE, aiming at evaluating students’ skills in a simulated clinical setting and providing them with feedback about their performances [38]. 

The identified studies were briefly described below, along with methodology details and main findings, as well as presented in Table 2 in chronological order.

Dobbie et al. [37] described the BELIEF teaching tool and cultural interviewing instrument, showing that 93.5% of the students succeeded in covering five out of six of its items. Additionally, 97% of the participants discussed the causes of the illness the patient believed she had 92% discussed the traditional beliefs in the treatment of the condition, 97% showed understanding of the impact the disease had on the patient’s life, and 94% acknowledged the emotional discomfort experienced by the patient. The empathy scale completed by the SP showed a score of over 90%. 

Rosen et al. [38] conducted tOSCE-based sessions with SPs, preceded by a theoretical introduction on cultural issues and the use of interpreters, and followed by a group discussion, showing an increase in students’ ability to assess patients’ culture and health beliefs. In total, 96% of the student participants considered the workshop successful and effective. Results showed that 92% of the students displayed a bigger understanding of a patient’s perception of illness. Additionally, 96% felt better prepared to elicit patients’ expectations of treatment. Then, 83% considered themselves more effective when asking about cultural issues, and 71% revealed a better understanding of their own cultural bias. 

Green et al. [39] presented the results of semi-structured interviews with the students on their perspective on the cultural content encountered during the OSCE station. The results revealed an overall successful identification of the learning goal of the station, concerns about its content contributing to stereotyping, and the separateness of the station from the other OSCE stations as being problematic. The appreciation of the feedback by the faculty observers and SPs was varied, ranging from satisfaction to disappointment, with some students describing the feedback as helpful and the SP encounter as realistic, while others finding them non-specific and artificial. 

Miller and Green [5], presenting the results of the interviews from Green et al. [39], focused on the students’ performance, self-assessment and their reflections on the learning effects of the simulation as well as a potential significance of a cultural content OSCE station as an educational tool. In general, the cultural content OSCE station was viewed as the most challenging, and what is more, it emphasized to the students the significance of incorporating cross-cultural knowledge and skills in all clinical situations and, by revealing the gaps in knowledge and skills, showed the importance of further development and practice.

Bertelsen et al. [40] presented an annual selective course in a clinical clerkship named Global Health, aiming to provide the knowledge necessary to work in culturally diverse medical settings. The simulated encounters, which employed standardized patients, were preceded by classroom discussions with instructions for the simulations. Results revealed that 86% of the students rated the course as excellent, with case discussions and simulated exercises receiving the highest notes. In the students’ evaluation of the core competencies, the use of cross-cultural communication skills was very highly appreciated. Moreover, the participants emphasized the contribution of the course to their understanding of ethical issues while working with minority populations, perception of social and cultural factors influencing health behavior and needs, as well awareness of global health disparities.

Maar et al. [54] described nine simulated scenario sessions to evaluate the recognition of health disparities of the Indigenous population, such as access to healthcare. After it, 80% of the students felt better prepared to judge when a good rapport with a patient was established, 77% felt better prepared to respond to the clinical presentations of a minority patient, 75% learned to react appropriately to a patient’s emotions, 75% believed they could better understand the patients’ perspective due to identifying main social determinants of health, and 84% expressed a better understanding of culture and its influence on patients’ health. Additionally, 100% of the tutors believed that the students were able to view the problem from the patient’s perspective, knew how to build a good rapport with a patient, and gained an understanding of the role of culture in the perception of health, illness, and treatment. Moreover, 85% of the faculty members declared that the students knew how to respond to clinical presentations of an indigenous patient and deal with patients’ emotions. Finally, the students described the simulations as a very useful experience. 

Additionally, we identified a study by Morell et al. [55] in which students participated in a conversation with a Cherokee Indian woman complaining of excessive menstrual bleeding, who displayed numerous communication patterns typical to the represented ethnic group. The authors observed a considerable increase in the students’ awareness of cultural sensitivity and their realization of the relevance of cultural issues to patient care but presented no results to support their observations; hence, the paper is mentioned separately in the present study.

### 3.3. Studies on Nursing Students

Twelve articles focused on nursing students were identified during the review, including two papers that presented results from the same intervention [11,42]. Their sample sizes varied between 25 and 104 students. The majority of them used the pre–post study design to evaluate the effects of the presented interventions [6,11,12,33,41,42,43,49,50,53], with four of them using additional post-intervention measures to capture students’ satisfaction and perceived effectiveness of the intervention, among others [7,11,12,42]. Additionally, some articles utilized mixed methodology to collect both qualitative and quantitative data. Moreover, two of the papers presented the results from studies comparing the effectiveness of interventions with simulated patients to other teaching strategies [7,49]. Different tools were used in the studies, including surveys developed by the authors [41,43,52] and ones described in previous papers [6,7,11,12,33,42,49,50,53], with Jeffreys Transcultural Self Efficacy Tool (TSET) being the most common one [6,12,33,50]. TSET is an instrument developed to evaluate the confidence and skills of nursing students engaged in providing culturally sensitive care to diverse patients [56]. It is an 83-item survey assessing respondents on 3 separate domains: a 25-item cognitive subscale, measuring confidence about the knowledge of variables influencing healthcare; a 28-item practical subscale rating self-efficacy in interviewing diverse patients and ability to learn about differences; and a 30-item affective subscale, which relates to the awareness of attitudes, values, and beliefs [56]. The instrument has been frequently tested since 1994 and contains numerous reports of content validity, construct validity, criterion-related validity, etc. [56]. Although TSET was originally addressed to undergraduate nursing students, due to its universal principles, it is often applied to measure the confidence of other healthcare professionals engaged in cross-cultural interactions with patients [56]. 

The identified studies were briefly described below, along with methodology details and main findings, as well as presented in Table 3 in chronological order.

Ruth-Sahd et al. [41] used two scenarios to foster the skills of vital signs assessment, practice working in interdisciplinary teams with culturally diverse patients, and assess students’ cultural awareness and sensitivity. The students were assigned the roles of various members of a healthcare team, such as a nurse, a pharmacist, a doctor, etc. The results revealed that 69% of the participants increased their communication skills, and 87% noticed an enhancement in cultural awareness and sensitivity. 

Grossman et al. [33] invited American and Norwegian students to participate in a study with two cultural content simulation scenarios. It showed differences in all pre- and post-intervention results in cognitive, practical, and affective domains for the American students and only affective and cognitive improvements for the Norwegian group. The students’ responses on the perception of cultural awareness and ways to increase it revealed similarities between the two groups. Both teams stressed the importance of such issues as knowledge about the cultural background and ethnic origin, awareness of differences among people regarding their values and beliefs, etc. On the other hand, the notion of multilingualism was emphasized by the Norwegian students, while the American participants valued the importance of verbal and non-verbal communication. 

Ndiwane et al. [42] reported students’ cultural competency evaluation during an OSCE simulation session with SPs portraying an underrepresented patient population. The survey showed students’ satisfaction with learning, self-confidence in learning through SP scenarios, and the effects of SP scenarios on critical thinking. The students believed the SP session would prepare them for clinical practice and was an enjoyable and motivating experience. The participants appreciated the simulation mostly for the realism of the scenario and the opportunity to practice communication skills but wished they had been given feedback from the SP immediately after the encounter. The cultural assessment survey also revealed an increase in five of the seven knowledge domains after the exposition of a cultural OSCE interaction. 

Guvenc et al. [52] conducted research to determine the effects of communication barriers on the quality of healthcare during a course on the safety of medication. Debriefing sessions and the data collected after the simulation revealed that although the students were offered a 4-hour weekly professional and general English course for 4 years of their study program and most of them had learned English in secondary schools, nearly all of the participants felt inadequately prepared to work with an English-speaking patient. The comments from the students also showed the simulation experience contributed to the recognition of various emotions, such as anxiety and helplessness, as the students reported communication barriers posed risks to patient safety. On the other hand, positive emotions were described, such as self-confidence, which was gained while working in a safe and controlled environment provided by a simulation. All in all, improvement in the awareness of language use, cultural differences, including the use of body language, and cultural safety was reported by students. 

Ndiwane et al. [11] carried out research during a cultural content OSCE module to measure the knowledge of cultural diversity and biases of the nursing students during an encounter with a culturally diverse SP. The results demonstrated a significant increase in knowledge in six out of seven variables. However, the students’ awareness of the problems people of color face when acquiring healthcare access did not improve. Finally, the satisfaction survey showed the highest score for satisfaction with learning, followed by critical thinking and self-confidence in learning. The comments from the students indicated an increase in awareness about ethnicity and sexual orientation. 

Chung et al. [49] carried out research on two groups of students to compare the effectiveness of simulation and case-based learning methods in teaching cultural competency. The results indicated an overall improvement in students’ cultural competence assessment scores, significant only for the case-based learning group. On the other hand, the cultural awareness and sensitivity scores increased significantly in both groups after the intervention. 

Ozkara San [50] presented a study assessing transcultural self-efficacy (TSE) during two Diverse Standardized Patient Simulation (DSPS) scenarios. The pre- and post-test data were collected with the 83-item Transcultural Self-Efficacy Tool (TSET), which showed an overall increase in transcultural self-efficacy for all subscales. The post-test scores indicated the greatest increase in the cognitive subscale, followed by the practical and affective ones. Two factors, marital status, with a change in the total TSET results, and religious preference, showing a difference on the affected subscale, had an impact on the students’ TSE results. 

Unver et al. [53], in a pre–post design study, aimed to assess language barriers and intercultural sensitivity. Intercultural Sensitivity Scale (ISS), comprising five subscales, i.e., engagement, respect, confidence, enjoyment, and attentiveness, was used before and after the scenario along with Intercultural Sensitivity Assessment Checklist, which assessed students’ performance during the encounter. The results on the ISS increased in all five subscales and showed no statistical significance. The results of the students’ performance revealed that all the students respected cultural values, and nearly all of them felt nervous when communicating with culturally diverse patients. The majority of the participants enjoyed or partly enjoyed communicating with culturally distinct patients, and all the students valued their patients’ opinions. 

Byrne [7] conducted an intervention with SP to evaluate the students’ level of cultural competence based on five individual constructs, i.e., awareness, knowledge, skills, encounters, and desire. The students were divided into a group that received only a cultural content lecture and one that received the lecture, followed by a simulation with SP representing a diverse patient population. No significant differences were noted between the lecture only and the lecture plus simulation groups. The students valued the SP experience as an opportunity to practice interviewing and interacting with a patient from a diverse cultural background and recommended sessions with SPs for their future educational practice. 

Nimmo et al. [12] used a standardized patient simulation-based learning experience (SBLE) to evaluate the level of cultural sensitivity and cross-cultural confidence in communication with rural Spanish-speaking patients. The Transcultural Self-Efficacy Tool (TSET) scales were administered before and after the simulation. The post-test mean results were significantly higher than the pre-test mean scores for all three domains. Finally, the comments from the students after the simulation indicated a very positive attitude toward the learning modality used in the study. The participants valued the opportunity to engage in interviews with a diverse population of patients, to gain knowledge of their culture and belief systems, and to identify their own weaknesses in cross-cultural communication skills, including the inability to speak a second language.

Turkelson et al. [6] conducted research to evaluate cultural competence, communication skills, and empathy through a standardized patient simulation-based learning experience (SBLE), which involved an encounter with a Spanish-speaking patient accompanied by a relative who was fluent in English. The Transcultural Self-Efficacy Tool scale, the Jefferson Scale of Empathy as well the Rural Characteristics Tool were used. The post-test scores were significantly higher than the pre-test results for all three domains of TSET, as discussed in Nimmo et al. [12]. No change was revealed on the Jefferson Empathy Scale. Finally, the results of the Rural Characteristics Tool, which focused on the assessment of students’ knowledge to provide rural healthcare, were of no relevance to the present review. 

Plaza del Pino et al. [43] carried out research in the context of a simulation with an actor portraying a migrant Moroccan patient. Semi-structured interviews conducted before the intervention revealed students’ insecurity and nervousness, mostly related to their lack of experience, language barrier as a communication inhibitor, difficulty with dealing with customs and daily routines of the migrant patient, and lack of adequate training on how to care for culturally diverse patients. The students’ answers to post-simulation questions showed their satisfaction with the ability to communicate effectively despite the language barrier and adapt different communication strategies, such as drawings of food, repeating words, etc. On the other hand, it proved difficult for the students to adapt their actions to the patient’s diverse cultural behaviors. However, the students admitted to acquiring new skills and knowledge about Muslim culture and succeeded in expressing empathy and understanding of the patient’s situation as a migrant. Finally, the participants realized that the language barrier was not the only obstacle in communication, as was expected before the simulation, but the lack of adequate cultural competences turned out to be equally significant.

### 3.4. Studies on Students from Other Faculties

The review revealed five papers on students from other than medicine and nursing healthcare faculties: three studies on dentistry students [4,35,46], one on pharmacy students [47], and one on physical therapy students [48]. Their sample sizes ranged from 48 to 155 students. Three studies used pre–post study design to measure the results of the interventions [35,47,48], with one choosing a retrospective pre- and post-test measurement to allow for better detection of the improvement in the students’ knowledge due to a phenomenon described as ‘response shift’ [35]. Two papers presented the results of post-test measures [4,46], and two of them used additional post-intervention measures to assess students’ satisfaction with the course [35,46]. A variety of tools was used in the studies, including surveys presented in other papers [35,46,48] and ones developed by the authors [4,47]. Finally, two of the studies aimed at comparing students’ performances with different educational strategies [47,48].

The identified studies were briefly described below, along with methodology details and main findings, as well as presented in Table 4 in chronological order.

Broder and Janal [46] conducted research to assess the communication skills of dental students during culturally enhanced training encounters with SPs, here referred to as patient instructors PI. The interventions were conducted in two separate sessions over a 9-month period. Following the interviews with patients, the Arizona Clinical Interviewing Rating Scale (ACIR) was completed by the SPs. A total of eight evaluations were performed in both rotations. The analysis of the mean scores during each of the two training sessions showed an overall increase with each round of the session, indicating a growing beneficial effect of the course on the students’ communication performance. The assessment score of the sessions made by the students was 4.4 out of 5 and was slightly higher for the second session. The students valued the feedback from the SPs (4.5), considered the training appropriate for their development (4.5), and appreciated the educational value of the experience (4.4). Moreover, the written comments from the participants stressed the educational value of the experience, which provided an opportunity to learn from real-life simulated interviews. The need for the continuation of the program was expressed by the students.

Wagner et al. [4] investigated the effects of three simulation-based sessions with SPs, known here as patient instructors PI, on the communication skills of dental students. Individual content checklists completed by the SPs revealed the good overall performance of the students. Finally, the analysis of content checklist items resulted in the identification of medical, dental, psycho-social, and cultural categories. The last item revealed that the students had no difficulties helping the patients resolve insurance issues or financial worries related to their immigration status. However, language barriers, inability to offer an interpreter’s support, a feeling of helplessness when discussing traditional healing methods, and failure in offering support systems, such as inviting a family member to accompany the patient, revealed weaknesses in creating successful culturally competent communication.

Wagner et al. [35] carried out research to assess dental students’ changes in attitudes and behaviors towards diversity and evaluate their satisfaction with a simulation-based learning program with SPs, known here as patient instructors PI. Following the two rounds of simulations, a retrospective pre-test and post-test, as well as a satisfaction survey, were carried out. A cross-cultural training evaluation tool designed by Welch [57] was administered to assess retrospectively the students’ attitudes and behaviors related to diversity. The mean score for both rotations was 4.4 on a 6-item diversity attitude scale. Moreover, the analysis from the two sessions revealed a significant impact of the simulation learning modality on the students’ attitudes, with the biggest improvement in the first round. Finally, the results from a satisfaction survey demonstrated a very positive reception of the program, with the highest satisfaction expressed during the first rotation.

Sales et al. [47] presented a study with pharmacy students divided into three separate intervention groups, i.e., a lecture group, a case scenario group, and a simulation group, to assess and compare cultural competence. A cultural assessment survey used before and after the interventions revealed that the three learning modalities enhanced the cultural competency of the students to different degrees and that none of the interventions increased the scores for all six examined domains. Cultural skills regarding modification of the interview during a diverse encounter improved most significantly in the simulation group and the lecture group. The simulation group showed a marked improvement in the cultural skills component regarding asking a patient about cultural preferences, cultural awareness item stressing mastery of cultural competency, and cultural empathy component regarding showing concern for cultural preferences. The ability to show concern for cultural preferences was also enhanced in the two other intervention groups. The cultural desire subscale revealed the simulation group students’ willingness to learn about other cultures, which fell in the case-study group and remained relatively steady in the lecture group.

Paparella-Pitzel et al. [48] conducted research to compare the cultural competence of physical therapy students divided into three groups, each employing distinct learning strategies, i.e., a lecture, an additional advanced lecture combined with role-playing and an advanced lecture followed by tOSCE simulation with SP. The mean pre-test and post-test scores demonstrated an improvement in competence levels across the three groups, with the highest post-test scores in the lecture plus role-play group. No significant differences were observed between the educational interventions. The authors emphasized, however, a very small sample of the enriched educational groups as compared to the control class, which might have influenced the results. Finally, the study revealed that no student reached a culturally ‘proficient’ level. 

### 3.5. Studies Conducted on Interprofessional Groups of Students

Four studies on mixed-group students were identified [9,44,45,51], including two articles that revealed results from the same intervention [44,45]. These included students of medicine, nursing, dentistry, pharmacy, dental hygiene, and dental therapy, occupational therapy, and dietetics. Their sample sizes varied from 175 to 45 students. All of the studies used the pre–post study design to assess the effects of the interventions presented in the papers, with three of them utilizing additional surveys to evaluate the students’ perceptions of the effectiveness of training sessions [9,44,51]. The assessment tools used in the studies included surveys employed in previous papers [9,51] as well as ones designed by the authors [44,45].

The identified studies were briefly described below, along with methodology details and main findings, as well as presented in Table 5 in chronological order.

Min-Yu Lau et al. [9] conducted research to test the viability of the Cultural Respect Encompassing Simulation Training (CREST) program, which attempted to provide students from rural and remote parts of Australia with strategies to communicate with culturally and linguistically diverse patients. Cultural competency was measured before and after each of the six simulations with SPs, delivered face-to-face via video link-up in three rural academic settings and one regional hospital. The results showed an increase in four out of five subscales, with the most visible improvement in cultural skills and the lowest scores in the cultural desire domain. The learning experience with the clearly presented and relevant subject matter, helping the students with communication and understanding diverse populations, was highly valued by the students. The simulations with SPs, who were described as very authentic by the participants, improved the students’ understanding of the importance of culture in healthcare encounters and were, therefore, highly appreciated by the learners.

Quick et al. [44] carried out research to assess cultural awareness and communication skills when caring for a limited English proficiency (LEP) patient. The pre- and post-training surveys revealed significant differences between the results for all seven survey items. For example, 80% of the participants rated their familiarity with practices while collaborating with LEP patient and interpreter as high after the training, compared to 10% before the intervention and 23% in the control group. Lastly, in the qualitative assessment, the students emphasized the importance and usefulness of the program, indicating that competent and comfortable work with interpreters requires efficient training. 

Woll et al. [45] described the Working with Interpreters as a Team in Health Care education program for oral health students that used simulation-based learning to enhance and assess cooperation with an interpreter and familiarity with health disparities experienced by LEP patients. The pre- and post-test results from the two sessions, which engaged separate groups of students, demonstrated a significant increase in the students’ skills to cooperate with interpreters in order to provide quality services to LEP patients. Lastly, their confidence also changed following the interventions, with 94% of the students feeling high competence after the simulation-based education. 

Garvey et al. [51] carried out research to assess the cultural capabilities and interprofessional attitudes of nursing, occupational therapy, and dietetics students and to evaluate students’ satisfaction with Tag Team Simulation TTS, an innovative teaching approach derived from theatre and allowing active engagement of a large group of people and interprofessional training. The participants’ performance was assessed with the Cultural Capability Measurement Tool (CCMT) and Interprofessional Attitude Scale (IPAS). The results revealed higher post-simulation scores for both measures. The scores on individual items demonstrated the growth of respect, communication, safety, quality, and teamwork. Furthermore, more than half of the students found the simulation intervention ‘extremely useful’ and ‘very useful’ in enhancing their cultural capabilities and interprofessional learning. Finally, the qualitative analysis of the participants’ responses showed a positive view of the simulation-based program, which was valued for its authenticity and meaningfulness. 

## 4. Discussion

The purpose of this systematic review was to collect and examine the evidence for simulations with SPs as a learning intervention to enhance the cultural competence of future healthcare professionals. It is worth emphasizing that cultural competence is a continuous process and should never be viewed as an end-point event [58]. What is more, it has been advocated in the literature that the term cultural humility rather than a traditional notion of cultural competence should be used in the context of cross-cultural medical training to indicate a lifelong commitment and an ongoing process of increasing cultural knowledge, self-reflection, recognition and respect for different cultural norms and practices [59]. The aims of the presented studies included the assessment of various areas of cross-cultural interactions, including cultural humility, knowledge, awareness, empathy and sensitivity, evaluation of interviewing skills and ability to establish a good rapport with culturally diverse patients, measurement of the skills during interpreter-supported interviews, assessment of familiarity with health disparities, estimating biases, evaluation of the ability to develop successful communication based on the understanding of cultural health beliefs, overcoming language barriers and adapting care to the culture of a patient. In the studies included in the present review, the authors used various standardized tools [6,7,9,11,12,33,35,42,46,48,49,50,51,53] as well as developed their own surveys [4,5,35,37,38,39,40,41,43,44,45,47,52,54] to evaluate the effectiveness of the study interventions. The majority of the results of the presented studies showed that simulations with actors portraying culturally diverse patients helped to achieve improvement in cultural competency and received positive ratings from the students, who expressed high satisfaction with the usefulness, relevance, meaningfulness, and effectiveness of the simulations, valued them for the realism of the encounter and/or expressed desire to participate in sessions with SPs in the future [9,11,12,35,39,40,42,43,44,46,51]. The authors of four studies aiming at comparing different educational strategies demonstrated no significant differences between them and found no evidence to support SP simulations as a more effective strategy for teaching cultural sensitivity [7,47,48,49]. Moreover, in a study by Chung et al., the students from a case-based group and not a simulation experience group achieved the most significant improvement in cultural competence following the intervention [49]. As noted by the authors, this may stem from the fact that the students in the case scenario group assessed their competence without putting it into practice, as it was in the case of the simulation group, who were given an opportunity to reflect on their own mistakes during a real rather than theoretical encounter [49]. Nevertheless, a noteworthy increase in self-reported confidence of the students, who expressed positive reactions to a simulation learning experience, was observed. What is more, the improvement in the level of different domains as a reaction to a different intervention, as demonstrated by Sales et al. [47], may indicate that the integration of more cultural learning opportunities into medical education based on a combination of teaching methods would be the most effective way to enhance cultural competency.

The simulation sessions presented in the studies attempted to generate real-life, authentic settings for the students to achieve their educational goals. This was possible due to the development of culturally competent simulation scenarios, enhancement of environmental fidelity by introducing specific props unique to a particular cultural group, employment of well-prepared actors to serve as patients, and creation of an active interprofessional learning experience. Firstly, many socio-cultural issues were introduced into the scenarios to create cross-cultural challenges for the students. SPs were, for instance, instructed to speak little English [6,40], or actors of distinct cultural backgrounds and languages were selected [4,6,7,9,12,37,39,42,51,52,53,54], religious belief systems continued to influence the patients’ attitude to treatment [4,33,37,38,41,43,46,47,49,50,53], treatment non-adherence stemmed from various socio-cultural factors and/or beliefs in the traditional healing power of nature [5,37,38,39,46,47,49], family members demanded to make decisions for patients [38], patients requested to be seen by a physician of the same sex [47,53,54], the choice of treatment strategy remained under the influence of language barriers, non-verbal communication behaviors [4,5,6,9,12,33,35,38,40,42,43,44,45,48,52,53], immigration, insurance or financial status [4,5,6,35,38,39,43], etc. Secondly, many authors emphasized manipulation of the physical environment as a means to achieve a realistic encounter by incorporating into the scenario’s various cultural artifacts, such as religious clothing, for example, hijab or abaya, wigs, ethnic-oriented jewelry, [33,49], etc. Next, the studies presented careful selection and training of the SPs, who were performers recruited from local talent agency [11], local indigenous health educators or actors [51,54], students from translating and interpreting faculty [44,45], faculty graduate students [47], members of local communities [4,6,9], family members [6], etc. It should also be noted that the performance of the patient actors was often highly valued by the students, who described them as highly authentic and convincing [54]. Finally, as a learner-centered tool, simulations with human actors helped to promote a dynamic learning environment to foster interdisciplinary awareness. In some studies, the realism of simulations was additionally enhanced by allowing interactions between healthcare professional teams and combining simulations with an interprofessional learning experience, which has facilitated both interprofessional and cultural competence learning [41,44,45,51]. Meanwhile, the readiness and need for the increased introduction of interprofessional education opportunities are presented by students and healthcare professionals [60,61]. Constructing cultural competence activities around the actual lives of groups of people and their identities, however, may lead to simplifications and stereotyping of those groups. The participants of the study in Green et al. and Miller and Green [5,39], for example, expressed concerns about the content of a simulated encounter with a minority and immigrant patient, who was depicted as a low-income and low-literacy woman, which could promote stereotypes about her people [5,39]. Therefore, it seems that the construction of simulation exercises should be based on avoiding group-oriented generalizations and the use of cases that go beyond stereotypical thinking about race, ethnicity, religion, etc.

Noticeably, the majority of identified studies involving simulations with SPs as a learning intervention to improve cultural competence were carried out with the students of nursing faculty [6,7,11,12,33,41,42,43,49,50,52,53]. Moreover, 67% of the articles with the use of nursing students were written in the last 5 years [6,7,11,12,43,49,50,53], compared to 17% of the papers on medical students [54] and 33% of research on other faculties [44,45,51] published in the same period of time. It may indicate a niche in the current state of knowledge on the topic and the need for more recent studies on the subject matter from the perspective of other faculties.

One of the principal parts of cultural competence is the knowledge of the language [13], and miscommunication due to language barriers might severely jeopardize the quality of healthcare [52]. This review demonstrated that the majority of the studies recognized linguistic barriers as obstacles to providing adequate cross-cultural healthcare [4,5,6,9,12,33,35,38,39,40,42,43,44,45,48,52,53]. The importance of learning a second language was stressed by Guvenc et al., who demonstrated the relationship between the insufficient command of English and the feeling of anxiety and stress experienced by Turkish students caring for an English-speaking patient [52]. The participants of a study by Unver et al. also stressed that the command of the second language was a groundwork for effective communication during cross-cultural medical encounters threatened by language barriers [53]. Finally, the Norwegian students in a bi-national study by Grossman et al. emphasized the importance of multilingualism when providing culturally competent care [33].

It was indicated in the papers that one way of dealing with language barriers in cross-cultural encounters is the use of interpreters. The significance of effective collaboration with interpreters was demonstrated in the studies by immersing interpreters into simulated scenarios but, most importantly, by providing training about the techniques of how to use an interpreter to overcome language barriers during a medical encounter. Bertelsen et al. developed one of the seven simulation scenarios around the Limited-English-Proficient (LEP) patient who needed the assistance of an interpreter [40]. Wagner et al. showed that the most frequently failed cultural item on the checklist was offering an interpreters’ help to a patient [4]. Min-Yu Lau et al., introducing Culturally and Linguistically Diverse CALD patients, evaluated the skills of effective work with an interpreter and noticed their improvement following the program, which included immersive simulation scenarios with SPs recruited from various ethnic and linguistic groups [9]. Woll et al. and Quick et al. measured the confidence level of the students engaged in communication with the use of an interpreter and aimed to evaluate how they are prepared to work with spoken-language interpreters by employing translating and interpreting students to participate in the scenarios [44,45]. Turkelson et al. invited a family member with a good knowledge of both English and a native tongue to accompany an LEP patient [6]. Then, training modules about effective collaboration with interpreters were offered to the students. The CREST program presented by Min-Yu Lau et al. involved a session on effective communication when English proficiency was not sufficient [9]. Quick et al. presented training modules on managing the care for LEP patients and using an interpreter [44]. Rosen et al. offered an instruction video demonstrating baseline skills of collaboration with interpreters followed by role-play practice [38]. Similarly, brief instructions on the effective use of interpreters in clinical situations were presented by Paparella-Pitzel et al. [48]. Moreover, not only were the students offered instructions on how to manage an interpreter during a medical encounter, but they were also familiarized with cultural, social, economic, and linguistic variables affecting the health of the diverse groups. Nimmo et al. engaged the learners in teaching modules about the culture and language of the LEP patient [12]. Similarly, basic Spanish online course and cultural information were offered to the students in a study by Turkelson et al. [6]. Woll et al., in collaboration with the students from the translation and interpreting faculty, presented an orientation cultural and interprofessional teamwork training to discuss LEP disparities and strategies to use an interpreter effectively [45].

All in all, although Ndiwane et al. demonstrated no increase in the knowledge about the second language in the cultural assessment survey [42], Rosen et al. noted no improvement in the skills of collaboration and communication with interpreters [38], and the students in Plaza del Pino et al. felt relieved when the linguistic barriers were not as detrimental to communication as they had expected [43], the high number of authors presented in this study who focused on the linguistic component of culture suggests that teaching the students how to communicate with patients in language-discordant situations seems highly relevant for cross-cultural education.

## 5. Conclusions

The literature examined in the present paper indicates that with the growing diversity of patient populations, the need for patient-centered care is increasing, and appropriate training that would equip future health professionals with skills to facilitate culturally congruent care is necessary. The studies have generally proven the usefulness of SP simulations for cultural competence training and indicated that simulated patient-based learning could enhance traditional teaching modalities, such as lectures, case-based learning, etc. Although the literature review identified the scarcity of studies using simulations with patients representing underserved populations in research on medical students, it managed to demonstrate an appreciation of the learning method among students of all medical professions. The study participants have emphasized their growing awareness of health disparities and understanding of the role of culture in the process of shaping the health of a population, which, in turn, resulted in an increase in their confidence. To conclude, we believe that more opportunities should be offered for future doctors, nurses, dentists, pharmacists, physical therapists, and other professionals to learn how to provide culturally sensitive care. Engaging professionally trained actors able to portray patients from different cultural backgrounds and provide competent and efficient feedback helps to successfully guide students through cultural education.

## Figures and Tables

**Table 1:** A systematic review question formulated according to the PICOS format.

PICOS Terms	Applied Inclusion and Exclusion Criteria
Population	Inclusion: Undergraduate students of medical and healthcare faculties Exclusion: Other participant groups, including postgraduate students or residency programs
Intervention	Inclusion: Studies describing and evaluating educational interventions with the implementation of the simulated patient method Exclusion: Interventions using methods other than simulated patients (e.g., simulations with mannequins, student (peer) role-plays, pre-recorded videos, virtual reality, or simulation games), non-interventional studies (e.g., tool validation only), and studies where simulated patients were used only to assess students’ pre-existing skills and not as part of the evaluated intervention involving SPs
Comparison	Any comparator interventions
Outcomes	Inclusion: Participating students’ levels of cultural competencies, cultural sensitivity or cultural humility, confidence, empathy, interviewing skills, also during interpreter-supported interviews, overcoming language barriers, adapting care to cultural needs of patients, participants’ satisfaction Exclusion: Not relevant to cultural competence, cultural sensitivity, or cultural humility
Study type	Inclusion: Original articles published in English matching the above-mentioned inclusion criteria Exclusion: Articles published in languages other than English

**Table 2:** Descriptive summary of studies on medical students.

Author, Year	Participants	Methods and Tools	Scenario Outline	Relevant Findings Related to Cultural Competences
Dobbie et al. 2003, [37]	200 first-year medical students (197 students participated in the interview with SP).	A description of the BELIEF teaching tool and a study assessing the interviewing skills. Before the simulation, the students participated in a course on health beliefs and group discussions as well as sessions with standardized patients.	A Hispanic female with reflux and abdominal pain who believes the pain is the result of a curse and tried traditional healing remedies unsuccessfully.	The results following a series of interventions, including sessions with standardized patients, were as follows: Belief: 97% Explain: 95% Learn: 92% Impact: 97% Empathy: 90% (assessed by SP) Feelings: 94%
Rosen et al., 2004 [38]	32 third-year medical students (24 completed the satisfaction survey).	A pre–post evaluation of a workshop, including a theoretical introduction and tOSCE sessions with SPs. Surveys assessing students’ attitudes and skills were filled out before and six weeks after the intervention. Students also filled out a self-evaluation survey immediately after the intervention.	1. A Japanese patient with stomach cancer and his daughter demanding to speak with a doctor about her father’s diagnosis. 2. An Ethiopian immigrant to Israel diagnosed with HIV. 3. A Chinese woman who, after trying traditional Chinese healing methods without success, wants treatment from a Western doctor. 4. An Orthodox Jewish woman with symptoms possibly related to sexual abuse by a family member in the past. 5. A Bedouin man with asthma refusing to quit smoking. 6. An illegal Bulgarian laborer with asthma.	Significant improvement in regard to health-belief assessment, sexual history taking, biopsychosocial interviewing skills, breaking bad news, and approach to treatment. No significant changes were observed in communication with family members and working with an interpreter. Overall satisfaction with the course in the post-intervention survey: 96% considered the course effective, 96% noticed improvements in their ability to elicit treatment expectations, 92% understood patient’s perception of illness, 83% inquired about cultural issues, and 71% understood their cultural bias.
Green et al., 2007 [39]	22 second-year medical students	Post-intervention semi-structured interviews with students on their perspectives on the SP encounter during a cultural competence OSCE station.	A Dominican woman with poorly controlled hypertension.	Identified themes covered learning goals, logistical issues, faculty feedback, and SPs. The overall students’ reception was positive. Some students pointed out that the separated format of the station posed a risk of promoting stereotypes and marginalizing the topic. SPs’ performance was mostly regarded as realistic and the feedback good, although sometimes too little specific.
Miller and Green, 2007 [5]	22 second-year medical students	Post-intervention semi-structured interviews with students on their perspectives on the SP encounter during a cultural competence OSCE station.	A Dominican woman with poorly controlled hypertension.	6 students (27%) were satisfied with their performance, 13 students (59%) realized gaps in knowledge and skills, 3 students (14%) were dissatisfied. Students’ reflections focused, among others, on inquiring about the reasons for non-adherence, patient’s perspective on the illness and social context, and exploring social and cultural factors associated with non-adherence.
Bertelsen et al., 2015 [40]	33 medical students	Analysis and evaluation of a 4-week Global Health selective course. Assessment of students’ performance mostly using direct faculty observation and feedback. Evaluation of the curriculum using surveys with closed and open questions.	1. A woman from rural Liberia in an obstetrical emergency. 2. A patient from Peru needing TB management. 3. A patient from Ghana and/or the Democratic Republic of Congo with hypertension. 4. A Chinese patient requiring counseling on smoking cessation. 5. An Ecuadorian diabetic patient. 6. A patient from the Democratic Republic of Congo with physical and psychological trauma. 7. A LEP patient needing the assistance of an interpreter.	Positive faculty and student feedback from the course, with 86% of students rating it as excellent. Highest ratings received by case discussion and experiential learning simulation. Increased students’ cross-cultural communication skills, ethical issues understanding in working with underserved populations, and appreciation of socio-cultural determinants of health-related needs and behavior, among others.
Maar et al., 2020 [54]	39 first-year medical students	Pilot evaluation study of 9 Simulated Cultural Communication Scenarios using Likert scale questions and open-ended questions on faculty and students’ experiences and perceptions.	1. A non-compliant female Indigenous diabetic patient uncomfortable with a male physician. 2. A male Indigenous patient with diabetes. 3. A male Indigenous patient with diabetes living under a lot of stress. 4. Tribal Indigenous police officer with frostbitten ears. 5. A male Indigenous patient who developed frostbite on his left hand, reluctant to receive help. 6. A male Indigenous patient with frostbitten fingers. 7. A male Indigenous patient recovering from addiction and living under a lot of stress needs a BP check-up. 8. A male Indigenous patient who is a single parent living with his sick mother and needs a BP check. 9. An Indigenous patient who travels a lot and needs a BP checked.	Improvements in students’ knowledge, skills, and understanding were believed to occur both by students and the faculty. Identified themes from qualitative data show the intervention’s potential to acquire culturally safe clinical skills due to the rich-in-context, authentic, safe dialogue with the patient.

**Table 3:** Descriptive summary of studies on nursing students.

Author, Year	Participants	Methods and Tools	Scenario Outline	Relevant Findings Related to Cultural Competences
Ruth-Sahd et al., 2011 [41]	73 nursing students	Pre-simulation inquiry with open-ended questions on students’ feelings about it. Post-simulation Likert scale survey on students’ feelings about its value.	Two simulated patient scenarios: 1. A Hispanic woman after abdominal surgery. 2. A Haitian Jehovah’s Witness patient after limb surgery.	Improved cultural awareness and sensitivity according to 87% of students.
Grossman et al., 2012 [33]	73 nursing students (48 Americans and 25 Norwegians)	Pre–post study using the Transcultural Self Efficacy Tool (TSET) measuring self-efficacy with subscales relating to cognitive, practical, and affective scores.	Two scenarios: 1. Somalian and Muslim patients for Norwegian students 2. Muslim and Italian Catholic patients for American students.	Increased self-efficacy in three TSET subscales and the total score (not significant only for the Practical subscale in the case of Norwegian students).
Ndiwane et al., 2014 [42]	29 nursing students	Pre–post study in the OSCE context using the Cultural Assessment Survey with questions on students’ awareness and knowledge. Additionally, adapted Student Satisfaction Survey was used after the intervention to measure students’ satisfaction, self-confidence, and the intervention’s effect on critical thinking. Pre-OSCE didactic presentations were held for students on cultural assessment and practices of selected populations. Post-OSCE video recordings of their own and reference performances were provided to students for comparison.	Two scenarios for each student: 1. A pregnant Latina patient or a Latina patient with diabetes 2. An African-American patient with hypertension.	Significantly increased knowledge in 5 out of 7 variables of the Cultural Assessment Survey. No changes observed in regard to the opinion variables. No change in knowledge about the language. Mean scores of Student Satisfaction Survey (Likert scale 1–5): - Satisfaction—4.22 - Self-confidence—4.28 - Critical thinking—4.45
Guvenc et al., 2016 [52]	104 fourth-year nursing students	Mixed-methods study: - Qualitative data collected during semi-structured debriefing interviews - Quantitative data collected with authorial surveys administered after debriefing.	A non-Turkish-speaking pregnant woman with severe preeclampsia.	Raised awareness of the possibility of providing care to a patient who speaks English in 95.2% of students. Recognition of the need to improve their English by 98.1% and the need to learn about different cultures by 81.7% of students. Language use, cultural differences, and patient safety gains reported in the qualitative part.
Ndiwane et al., 2017 [11]	63 nursing students (51 completed surveys)	Pre–post intervention using the Cultural Assessment Survey with pre-test used to assess cultural competency knowledge, followed by cultural sensitivity presentation and an OSCE module with an SP that was video recorded and then given to the student along with a reference video for comparison. After that, a debriefing session was held, and later, students completed the post-test and Student Satisfaction Survey.	A male African American patient with hypertension.	Statistically significant increase in knowledge. Generally good reception by students with highest satisfaction scores, followed by critical thinking and self-confidence.
Chung et al., 2019 [49]	80 nursing students (38 simulation-based learning and 42 case-based learning)	Pre–post two-group study using the Cultural Competence Assessment Survey CCA, involving Cultural Awareness and Sensitivity scale CAS and Cultural Competence Behaviors CCB—(only pre-intervention).	A 60-year-old female Muslim needing a dressing change after hip surgery.	Improvement of CCA. Case-based learning group: 3.73 to 4.14; Simulation group: 3.98–4.02 Improvement of CAS. Case-based learning group: 5.94–6.09; Simulation group: 5.97–6.22
Ozkara, 2019 [50]	53 nursing students	Longitudinal pre–post intervention using the Transcultural Self-Efficacy Tool (TSET) and two other authorial tools: Simulation Survey and Simulation Participation Survey.	1. A 65-year-old female Muslim patient from Turkey after surgery. 2. A 55-year-old American patient of Irish and Italian origin who is a Methodist and self- identifies with the LGBTQ population, suffering from diabetes.	Overall increase in transcultural self-efficacy (TSE) in all 3 learning domains. Significant changes in the cognitive learning subscale followed by the practical and affective ones.
Unver et al., 2019 [53]	34 final-year nursing students	Pre–post model study with Intercultural Sensitivity Scale (ISS) and Intercultural Sensitivity Assessment Checklist aimed at evaluation of cultural sensitivity and communication barriers.	1. A Muslim Arabic- Palestinian male with diabetes, an immigrant to Turkey, fleeing the civil war in Syria, strictly following Ramadan principles and refusing to eat and take medicines, requesting a male nurse, speaks little Turkish. 2. A catholic White-American female patient with asthma and migraine, holidaying in Turkey, presenting at the emergency unit, speaks only English.	No significant change on Intercultural Sensitivity Scale (ISS). Intercultural Sensitivity Assessment Checklist revealed: Less than 15% were fully happy with the experience, half never collected information about diverse cultures, 41.18 did not feel confident, and 52.94% respected patients’ opinions. All students respect cultural values, 88.2% considered themselves open-minded nearly all (97.05%) respect different cultural behaviors.
Byrne, 2020 [7]	38 nursing students	Quasi-experimental mixed method study with the IAPCCSV^©^ used for pre–post and an open-ended survey for qualitative part on students’ perception of the intervention’s effectiveness. Comparison of two educational strategies—lecture only and lecture plus simulation with SPs groups.	A patient from a culturally diverse background seeking nutritional assessment and advice (including the use of cultural terms related to diet unknown to the students).	No significant differences between the lecture group and the lecture plus simulation group were observed. However, the students describe the interaction with SP as valuable, helpful, and highly recommended.
Nimmo et al., 2021 [12]	25 nursing students (results analyzed for 23 students)	Pre–post study using the Transcultural Self-Efficacy Tool (TSET). Qualitative data from student self-reflection assessments were also analyzed. Online learning modules with cultural content and basic Spanish lessons were offered before the simulation.	A Spanish-speaking patient from a rural Hispanic population. Focus of the simulation on the management of chronic disease, e.g., diabetes mellitus type 2	Significantly higher post-test results on all 3 TSET subscales. Positive reception by students describing the simulation experience as ‘helpful’ and ‘enjoyable’ and acknowledging its role in identifying their knowledge weaknesses.
Turkelson et al., 2021 [6]	26 nursing students (results analyzed for 23 students)	Quasi-experimental pre–post study using the Transcultural Self-Efficacy Tool (TSET), the Jefferson Scale of Empathy, and the Rural Characteristics Tool. Additionally, online modules dealing with culture, language, and communication strategies were introduced as pre-simulation education.	A Spanish-speaking Hispanic patient in a rural primary healthcare setting accompanied by a family member fluent in both Spanish and English. Three scenarios were developed, and they portrayed problems such as lack of insurance, lack of previous medical records, etc.	Significantly higher post-test results for all 3 TSET subscales. Substantially higher pre-test and post-test mean scores for the affective subscale in comparison with other scales. No significant pre–post changes were observed on the Jefferson Scale of Empathy.
Plaza del Pino et al., 2022 [43]	63 fourth-year nursing students (56 completed the interviews)	A pre–post qualitative descriptive study aimed at the evaluation of cultural competences when caring for a migrant patient. Semi-structured interviews were conducted before and after simulations. Instructions about the scenario were given to the students. Following the simulations, the students engaged in discussions and feedback from the professors.	A Moroccan Muslim patient admitted with hyperglycemia understands Spanish.	Satisfaction with the ability to communicate in a language-discordant situation, satisfaction with the use of various communication strategies, e.g., drawings. Students felt new skills and knowledge about different cultures were gained. Students were able to show empathy to the patient. They had difficulties adapting their actions to different cultures and customs. Realization of the importance of cultural competence training.

**Table 4:** Descriptive summary of studies on students of other faculties.

Author, Year	Participants	Methods and Tools	Scenario Outline	Relevant Findings Related to Cultural Competences
Broder and Janal, 2006 [46]	143 third and fourth-year dental students	Two-part clinical communication program—CC1 and CC2—with CC2 containing cultural and mental health sensitivity issues. Arizona Clinical Interviewing Rating Scale (ACIR) was used to rate students’ performance after each scenario. Additionally, a course evaluation survey was filled out by students.	Four scenario rounds were completed in each part. Exemplar CC2 scenario focused on cultural aspects: A 35-year-old Muslim female with osteogenic sarcoma.	Improvements in the ratings of students’ clinical communication were observed between training rounds. Positive ratings of the training sessions by students (mean item scores between 4.4 and 4.6 on a 1–5 scale).
Wagner et al., 2007 [4]	118 dental students (79 completed a third rotation)	Three rotations extended over junior and senior years involving clinical interviews assessed with the use of standardized rating scales and content checklists. Before the rotations, a lecture and seminar were presented to students on cultural aspects, among others.	The second and third rotations contain cross-cultural aspects, which involve such issues as language and religious differences, immigration status, culture-based beliefs about oral health, culture-based trust in alternative treatment, etc. Examples include: 1. A non-compliant patient with a heart murmur. 2. A smoking cessation case.	The overall performance was good, with improvements between encounters on a given day. Most frequently passed cultural items involved: patient confusion on insurance coverage and related financial concerns. The most frequently missed cultural items involved: language barriers, alternative healing strategies, access to care, and support systems.
Wagner et al., 2008 [35]	155 dental students	Retrospective pre–post evaluation study aiming at assessing students’ changes in attitudes and behaviors towards diversity was carried out during two cross-cultural rotations with SPs. A cross-cultural training evaluation measure developed by Welch was used to capture students’ diversity-related attitudes and behaviors. The students’ satisfaction was also assessed with a separate survey developed by one of the authors.	Patients from Central and South America, Eastern Europe, Africa, India, and Asia presenting challenging cultural contents such as belief in alternative healing methods, different worldviews and religious values, different attitudes towards health and healthcare, language barriers, issues related to insurance and immigration status, etc.	Retrospectively reported improvement in diversity-related behaviors and attitudes across the two rotations. Students’ high satisfaction with the course in terms of both usefulness and enjoyableness.
Sales et al., 2013 [47]	84 second-year pharmacy students (26 lecture-based learning, 30 case-based learning, 28 simulation-based learning)	Pre–post comparison study using cultural assessment surveys before and after three separate interventions—a 50-min lecture, case-scenario with a brief 10-min lecture, and simulation with SP with a brief 10-min lecture. The cultural assessment tool was used, supplemented with questions developed by authors.	Two SP encounters (students assigned into groups of 6) 1. A 72-Indian patient discharged from the hospital where he received treatment for DVT. In a hospital pharmacy, he refuses to pick up some of the medicines prescribed by a physician and admits to not planning to go to a follow-up cardiology unit appointment. 2. An anxious Muslim patient at the hospital refill clinic refusing to speak to a pharmacist who is of the opposite gender.	No activity managed to raise survey scores in all domains. Significantly bigger improvement in the cultural skills involving modifying communication style, demeanor, and interviewing questions among students from simulation and lecture than in the case-scenario group. After the intervention, simulation group students were more likely to express the desire to learn about the health beliefs and practices of different cultures and ethnic groups.
Paparella-Pitzel et al., 2016 [48]	37 second-year physical therapy students (25 standard lecture, 6 case-based enriched learning, 6 simulation-enriched learning)	Longitudinal, 2-year pre–post study comparing students’ levels of cultural competence at three time points in three different educational strategies—standard lecture, additional lecture along with a role-play session, or Teaching OSCE (TOSCE) with SP. The Inventory for Assessing the Process of Cultural Competence Among Health Care Professionals-Revised (IAPCC-R) scale was used.	Culturally diverse scenarios with SPs—no further information available.	No significant differences were observed between the educational interventions. In the total sample, significant score improvement was noticed between the pre-test and first post-test, and the pre-test to the second post-test, with a significant deterioration between the first post-test and the second post-test.

**Table 5:** Descriptive summary of studies on interprofessional groups of students.

Author, Year	Participants	Methods and Tools	Scenario Outline	Relevant Findings Related to Cultural Competences
Min-Yu Lau et al., 2016 [9]	45 nursing and medical students and junior practitioners	Mixed-methods evaluative study with cultural competency being measured before and after Cultural Respect Encompassing Simulation Training (CREST) videoconference sessions using a survey covering 5 domains of cultural competency and assessment of learning experience by the participants.	SPs from different ethnic groups, including Asia, Africa, the Middle East, Europe, and Aboriginal Australians.	Significant improvement was noticed in 4 out of 5 cultural competency domains—cultural skills, encounters, knowledge, and awareness. Increase in understanding of non-verbal behavior, respect for diverse cultures, and effectiveness of work with interpreters. Teaching quality and learning experience highly rated by students.
Quick et al., 2019 [44]	89 dental, dental hygiene, and dental therapy students (49 completed the surveys)	Pre–post study on students’ confidence and familiarity with best practices and talking to a limited English proficient patient and a patient with an interpreter. Qualitative data were collected during focus groups with participants. Comparison group involved data from 245 dental, dental hygiene, and dental therapy students who did not participate in the intervention.	41 translating and interpreting students served as SPs with scenarios focused on a limited English proficient patient and a patient with an interpreter.	Significant pre–post differences in participants’ self-ratings for all 7 survey items. Usefulness and relevance of the training concepts stated by students in focus groups.
Woll et al., 2020 [45]	175 dental, dental hygiene, and dental therapy students	Pre–post study on students’ confidence and familiarity with best practices and talking to a limited English proficient patient and a patient with an interpreter.	41 translating and interpreting students served as SPs with scenarios focused on a limited English proficient patient and a patient with an interpreter.	Significant pre–post improvements in all survey parameters, including respondents’ familiarity with provider and interpreter best practices, legal protections, safety issues, and health disparities experienced by LEP patients and their confidence with talking to a LEP patient and employing best practices.
Garvey et al., 2022 [51]	72 nursing, occupational therapy, and dietetics students (55 completed qualitative survey)	Pre–post study using the Cultural Capability Measurement Tool (CCMT) and Interprofessional Attitude Scale (IPAS). Post-simulation students also were asked open-ended questions on the experience and its impact.	An Aboriginal woman after limb amputation accompanied by her daughter and an Aboriginal health worker during hospital discharge.	Significantly higher post-simulation of the overall CCMT score and the respect, communication, safety, and quality subscales. Changes for advocacy and reflection subscales were not significant. More than 50% of students described the simulation experience in relation to their cultural capability learning and interprofessional education as ‘extremely’ and ‘very useful’.

## Data Availability

Not applicable.
